# Graph Neural Network Reveals the Local Cortical Morphology of Brain Aging in Normal Cognition and Alzheimer’s Disease

**Published:** 2026-01-23

**Authors:** Samuel D. Anderson, Nikhil N. Chaudhari, Nahian F. Chowdhury, Jordan Jomsky, Xiaoyu (Rayne) Zheng, Andrei Irimia

**Affiliations:** 1 Ethel Percy Andrus Gerontology Center, Leonard Davis School of Gerontology, University of Southern California, Los Angeles, CA 90089, USA; 2 Corwin D. Denney Research Center, Alfred E. Mann Department of Biomedical Engineering, Viterbi School of Engineering, University of Southern California, Los Angeles, CA 90089, USA; 3 Neuroscience Graduate Program, University of Southern California, Los Angeles, CA 90089, USA; 4 Department of Materials Science and Engineering, University of California, Berkeley, Berkeley, CA 94720, USA; 5 Lawrence Berkeley National Laboratory, Berkeley, CA 94720, USA; 6 Department of Quantitative & Computational Biology, Dana & David Dornsife College of Arts & Sciences, University of Southern California, Los Angeles, CA, USA; 7 Centre for Healthy Brain Aging, Institute of Psychiatry, Psychology & Neuroscience, King’s College London, UK

**Keywords:** Local Brain Age, Morphometry, Graph Neural Network, T1-weighted MRI

## Abstract

Estimating brain age (BA) from T1-weighted magnetic resonance images (MRIs) provides a useful approach to map the anatomic features of brain senescence. Whereas *global* BA (GBA) summarizes overall brain health, *local* BA (LBA) can reveal spatially localized patterns of aging. Although previous studies have examined anatomical contributors to GBA, no framework has been established to compute LBA using cortical morphology. To address this gap, we introduce a novel graph neural network (GNN) that uses morphometric features (cortical thickness, curvature, surface area, gray/white matter intensity ratio and sulcal depth) to estimate LBA across the cortical surface at high spatial resolution (mean inter-vertex distance = 1.37 mm). Trained on cortical surface meshes extracted from the MRIs of cognitively normal adults (*N* = 14,250), our GNN identifies prefrontal and parietal association cortices as early sites of morphometric aging, in concordance with biological theories of brain aging. Feature comparison using integrated gradients reveals that morphological aging is driven primarily by changes in surface area (gyral crowns and highly folded regions) and cortical thickness (occipital lobes), with additional contributions from gray/white matter intensity ratio (frontal lobes and sulcal troughs) and curvature (sulcal troughs). In Alzheimer’s disease (AD), as expected, the model identifies widespread, excessive morphological aging in parahippocampal gyri and related temporal structures. Significant associations are found between regional LBA gaps and neuropsychological measures descriptive of AD-related cognitive impairment, suggesting an intimate relationship between morphological cortical aging and cognitive decline. These results highlight the ability of GNN-derived gero-morphometry to provide insights into local brain aging.

## Introduction

The cortical surface undergoes significant changes during normative aging and plays a central role in revealing the pathogenesis of neurodegenerative disorders such as Alzheimer’s disease (AD) [1–3]. Changes in cortical thickness (CT) [4, 5], surface area (SA) [6, 7], sulcal depth [8, 9], gray/white matter intensity ratio (GWR) [10, 11], and cortical curvature [12, 13] are all hallmarks of brain aging. Deviations from normative patterns of cortical aging manifest early in AD and, in some cases, may even precede clinical symptoms [1, 2, 14]. As such, there is value in studying how cortical features vary with age in both cognitively normal (CN) and cognitively impaired (CI) populations. Chronological age (CA), however, does not adequately capture the heterogeneity of aging effects across individuals. In contrast, the biological age of the brain, i.e., brain age (BA), quantifies the progressive, cumulative changes in human anatomy that accompany advancing senescence [15, 16]. This allows measures of disease risk or progression to be grounded on the observed variances of aging profiles. The difference between BA and CA, termed BA gap (BAG), reflects advanced (BAG > 0 y) or delayed (BAG < 0 y) brain aging, and may serve as a candidate biomarker of excessive aging observed in neurodegenerative disorders such as AD [17–19].

Typically, BA is computed as a single global value, global BA (GBA), which summarizes aging across the entire brain [17, 20, 21]. While informative, this method requires regional differences in brain aging to be localized *indirectly*, often via saliency methods whose reliability and interpretability may vary [22–25]. In contrast to GBA, local BA (LBA) computes vertex- or voxel-level BA, yielding local BAGs (LBAGs) that *directly* capture spatial variability in aging across the brain. An LBA model generates a spatial map of the brain by utilizing measured attributes—such as local CT or magnetic resonance image (MRI) intensity—to produce cortical LBA maps. LBA naturally facilitates downstream analyses that relate *local* aging to functional or cognitive measures, thereby enhancing the utility and specificity of BA modeling in both research and clinical contexts.

Previous LBA models have been hindered by both feature selection and methodology. For example, existing models leverage MRI intensities derived from $T_{1}$-weighted ($T_{1}\text{w}$) MRI to quantify disease risk [26, 27]. While these intensities encode subtle molecular and structural interactions within brain tissue [28], their relationship to cortical morphology is complex [29]. This limits the interpretability of black-box deep learning models [30], as MRI intensity conflates multiple structural and molecular factors, making it unclear which aspects of cortical biology drive model predictions. Methodologically, existing LBA models have relied upon convolutional neural networks (CNNs) to extract aging patterns. CNNs have achieved great success across diverse tasks, but impose several key assumptions which may limit their applicability to BA estimation. Namely, CNNs draw inferences by dividing the brain into volumetric patches of arbitrary sizes and then analyzing their convolutional properties. This means that regions which are anatomically distant may be treated as topologically close by the model. Additionally, CNNs assume neighborhood regularity, which conflicts with the highly irregular geometry of the cortex [31–34].

Motivated by these limitations, we leverage a graph neural network (GNN) trained on surface morphometry instead of volumetric data. GNNs utilize graph-based representations to facilitate pattern recognition while preserving structural irregularities. Our approach conceptualizes the cortical surface, derived from $T_{1}\text{w}$ MRI, as a three-dimensional surface mesh, with morphometric features mapped to each vertex on this mesh. By preserving the geometric and topological continuity of the cortical surface, this approach enables anatomically faithful modeling of inter-regional dependencies and local aging trajectories. Deriving morphometric features from MRI intensities prior to BA inference improves model interpretability and facilitates the use of well-established explainability techniques—such as feature ablation or saliency—to explain the *specific* morphology underlying model decisions. Recent work has demonstrated that GNNs achieve state-of-the-art performance in surface-based GBA computation [35], and outperform alternative methods—such as CNNs and vision transformers—in image segmentation [36–38], where accurate delineation of fine anatomical boundaries is critical. However, few studies have examined GNNs in the context of morphometric aging [21, 35], and none have extended them to LBA. To address this gap, we introduce a graph U-Net [39] architecture designed to compute vertex-level LBA from cortical morphometric features. Collectively, these properties enable robust quantification of local aging, and provide a scalable foundation for studying individual variability in morphometric aging across both CN and AD populations.

## Methods

### Data.

$T_{1}\text{w}$ MRI scans were aggregated across multiple sources to enhance the generalizability of our model and findings. All participants provided written informed consent at their respective contributing institutions. This study was conducted in accordance with the U.S. Code of Federal Regulations (45 C.F.R. 46) and the Declaration of Helsinki. The training sample, used both to train our model and in cross-validation, was comprised of CN adults from the UK Biobank (UKBB), National Alzheimer’s Coordinating Center (NACC), and Information eXtraction from Images (IXI) datasets [40–42], totaling 14,250 scans spanning a broad CA range. Model evaluation was performed using the Alzheimer’s Disease Neuroimaging Initiative (ADNI) dataset, which included 1,129 scans from CN participants, and 477 scans from individuals with a clinical diagnosis of AD [43]. Participant demographics and dataset characteristics are summarized in Table 1.

The ADNI was launched in 2003 as a public–private partnership, led by Principal Investigator Michael W. Weiner, MD. The primary goal of ADNI has been to test whether serial MRI, positron emission tomography, other biological markers, and clinical and neuropsychological assessment can be combined to measure the progression of mild cognitive impairment (MCI) and early AD. For ADNI CN adults, inclusion criteria are: no memory complaints, a Clinical Dementia Rating (CDR) of zero, no significant impairment in cognitive function or activities of daily living, and a score of at least nine on the Logical Memory II subscale of the Wechsler Memory Scale–Revised. For NACC, participants were included in the training set only if physicians deemed them CN based on cognitive assessment and/or personal and medical history. Some participants contributed scans acquired at different time points. In the training sample, all UKBB and IXI participants contributed only a single scan, whereas NACC contributed 4151 scans across 3047 participants. To prevent data leakage, these additional scans were excluded from cross-validation but were retained as independent samples during training to mitigate dataset imbalance (particularly the predominance of UKBB). In the testing sample, the ADNI CN cohort comprised 1,129 scans from 517 unique participants, while the ADNI AD cohort comprised 477 scans from 354 unique participants.

### Preprocessing.

$T_{1}\text{w}$ MRI scans were preprocessed using FreeSurfer (FS) to extract high-resolution cortical surface features. These features served as inputs to our GNN model and included CT, sulcal depth, curvature, SA, and GWR. The FS pipeline included intensity normalization, skull stripping, bias field correction, and segmentation of brain tissue into white matter (WM), gray matter (GM), and cerebrospinal fluid (CSF). FS reconstructs cortical surfaces by initializing a tessellated sphere within the WM and iteratively deforming it outward toward the GM–CSF boundary, guided by local intensity gradients. This process ensures anatomically precise localization of cortical structures, and enables the extraction of those vertex-level features used in our model [44], yielding a single, unique cortical surface mesh per subject and hemisphere. To derive a single mesh per-subject, we concatenate the two hemispheres’ representations. This results in a disconnected graph where each hemisphere remains fully self-contained. To facilitate intersubject comparisons and reduce noise, cortical surfaces were aligned via spherical registration, which maps each cortical mesh to a set of standardized cortical atlases. This allows subject-specific cortical morphometry to be represented in atlas space, with multiple spatial resolutions being available [45]. We leverage these atlases to support information transfer across resolutions, enabling a pooling/unpooling strategy that is independent of training data, being instead guided by biological convention.

### Atlas resampling.

To enable information transfer across graph representations corresponding to different atlas mesh resolutions, we defined *receptive fields* for each vertex in a bottom-up manner, starting at the lowest resolution and proceeding upward. Euclidean coordinates were obtained for each vertex v at resolutions i and i+1 using Euclidean projections of each atlas. For each vertex in resolution i, we identified the closest vertex in resolution i+1 based on these coordinates, forming cross-resolution pairs $\left(v_{i},v_{i+1}\right)$. Using the atlas mesh, we then identified the 1-hop neighboring vertices of each $v_{i+1}$. The matched vertex $v_{i+1}$ together with its neighbors define the receptive field of $v_{i}$, denoted as $R\left(v_{i}\right)$.

We further define the inverse of the receptive field such that, for any vertex $v_{i+1}$, its inverse receptive field $R^{-1}\left(v_{i+1}\right)$ is the set of all lower-resolution vertices $v_{i}$ whose receptive field contains $v_{i+1}$. The inverse receptive field of a vertex $v_{i+1}$ can therefore be understood as a collection of all vertices $v_{i}$ which have $v_{i+1}$ in *their own* receptive fields:

$$R^{-1}\left(v_{\text{i}+1}\right)=\left\{v_{\text{i}}|v_{\text{i}+1}\in R\left(v_{\text{i}}\right)\right\}$$

**Figure 1.**
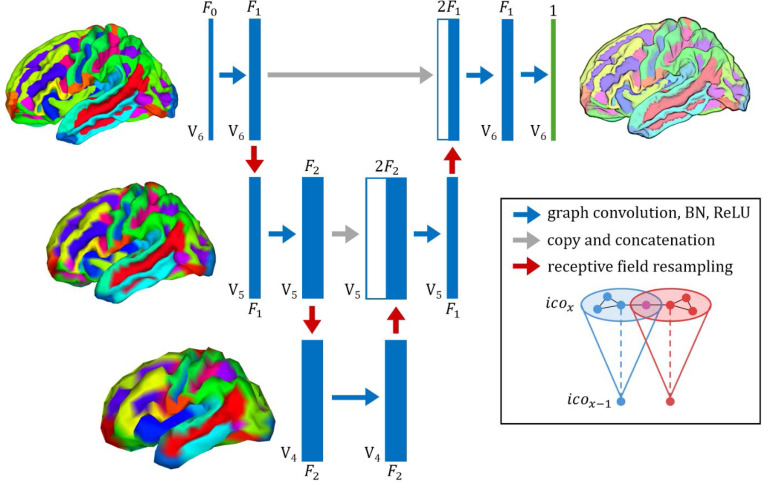
Graph U-Net architecture for cortical surface modeling. Blue rectangles denote feature maps defined on the cortical surface mesh at different atlas resolutions (e.g., V_4_, V_5_, V_6_). Joined blue and white rectangles represent skip connections, where features from earlier blocks are concatenated with those from later blocks. Each rectangle is annotated with the number of features F and vertices V, indicating mesh resolution. The final output (green box with cortical rendering) is a vertex-wise cortical map predicting age at each surface location. Note that the last block does not undergo batch normalization (BN) or contain a rectified linear unit (ReLU).

Downsampling is performed by averaging features within each receptive field, producing one representation per lower-resolution vertex. Conversely, upsampling is achieved by averaging across inverse receptive fields. Both operations yield feature matrices aligned to their respective resolutions, enabling smooth information flow across scales (Figure 1). In our GNN model, each resampled cortical atlas represents a sample-level graph. Mesh vertices function as graph nodes, with triangular mesh faces defining node connections that encode spatial adjacency within each hemisphere. Because all cortical surfaces are resampled to the same standardized atlas, these sample-level graphs differ only in the morphometric features of their nodes. Convolution-like operations aggregate information from neighboring nodes to learn localized feature representations, which are progressively refined across mesh resolutions via resampling. This process ultimately converges into a single feature per node, representing LBA per vertex.

### Model structure.

As shown in Figure 1, the model follows a hierarchical U-Net structure designed for cortical meshes. It consists of an encoding and decoding phase, operating across three FS atlas resolutions: ico6, ico5, and ico4. The number of vertices $V_{n}$ within each block is dependent on that block’s resolution: ico6 has $V_{6}=81924\;$ vertices, ico5 has $V_{5}=20484$ vertices, and ico4 has $V_{4}=5124$ vertices. With the exception of the initial block, which in our model used 5 morphometric features $\left(F_{0}=5\right)$, the number of features $F_{n}$ is a hyperparameter and can be modified *ad libitum*. Feature sizes of $F_{1}=8$ and $F_{2}=16$ were found to yield lowest validation losses. The output block always returns a single feature per vertex, representing LBA. In the *encoding phase*, graph convolutional network layers (GCNConv) [46] extract feature representations at each block. We then apply batch normalization (BN) [47] followed by a rectified linear unit (ReLU). The network progressively downsamples from ico6 to ico4 using unweighted downsampling layers, reducing spatial resolution while preserving essential structure. In the *decoding phase*, the network restores features to higher resolutions (ico4 to ico6) through unweighted upsampling layers. Skip connections between corresponding encoding and decoding blocks ensure that information from previous blocks is retained. GCNConv, BN, and ReLU are applied sequentially between each upsampling operation. In the final block, we only apply GCNConv. The model generates a final cortical representation at ico6, and is trained and tested using vertex-wise mean absolute error (MAE) as its loss function. Model training and evaluation were performed using a single NVIDIA A100 GPU. The model was trained for 50 epochs using a batch size of 128, and was optimized using the Adam optimizer [48] at a learning rate of 0.01. These parameters were selected empirically.

### Medial wall removal and smoothing.

After the model has completed training and generated predictions for the test set, we remove the medial wall from further consideration. We do this because the medial wall is not a part of the cortex and would distort topology if removed earlier. We next apply smoothing to the remaining predictions. This is done by averaging each node’s LBA with those of its neighboring nodes. In our approach, we included neighbors up to two steps away and repeated the averaging process four times, removing visually apparent model artifacts while retaining cortical variability.

### Integrated gradients.

To assess the contribution of each morphometric feature to our model’s predictions, we used integrated gradients (IGs). We opted for this method over alternative saliency methods due to its relative success in biomedical contexts [49, 50]. IGs work by comparing a subject to a baseline and generating N intermediate “pseudo-subjects” that gradually transition from the baseline to the actual subject. For each of these steps (we used the default N=50), IGs compute the local contributions (gradients) of each feature with respect to the model’s predictions, which they then combine. This yields per-subject maps of feature importance, which we averaged across subjects to identify group-level patterns. The resulting maps preserve relative scaling, allowing direct comparison of contribution magnitudes across features. We applied this procedure to both our ADNI CN and ADNI AD test sets. For CNs, we derived our baseline by setting all features to 0, corresponding to a z-score of 0 relative to the training set. For the AD group, we instead used a randomly selected batch of CN participants as baseline, which was kept fixed across all AD iterations [51]. This allowed us to identify AD-specific changes in morphometry that are relevant to LBA.

### Semi-global bias correction.

Previous studies have demonstrated that BA estimates exhibit systematic bias toward the mean of model training data, with younger individuals consistently overestimated and older individuals underestimated [52]. To address this, we applied a semi-global correction after smoothing. At each vertex, LBAG was regressed on CA [52, 53], producing a unique slope and intercept per vertex. These coefficients were then averaged across all vertices to obtain a single slope and intercept for the cortex, which were used to adjust each subject’s predictions based on their CA. This *semi-global* approach balances two extremes: a fully global correction, which fits a single regression using GBAGs, and a fully local correction, which adjusts each vertex independently. The former is vulnerable to subject-level outliers, while the latter may oversmooth biologically relevant variation.

Formally, let $LBA_{vs}$ denote the computed LBA for vertex v and subject s, and $CA_{s}$ the CA for subject s. The LBAG is defined as $LBAG_{vs}=LBA_{v,s}-CA_{s}$ and serves as the dependent variable. For each vertex, we regressed $LBAG_{vs}$ on $CA_{s}$ across subjects, obtaining a slope $m_{v}$ and intercept $b_{v}$:

$$LBAG_{vs}=m_{v}CA_{s}+b_{v}$$

This yields $V_{6}=81,924$ vertex-specific slopes $m_{v}$ and intercepts $b_{v}$. We then averaged $m_{v}$ and $b_{v}$ across all v to obtain the semi-global slope $m_{\text{μ}}$ and intercept $b_{\text{μ}}$. The averaged coefficients yield an adjustment term $m_{\text{μ}}CA_{s}+b_{\text{μ}}$ which we apply to every v for a given s. This removes CA-related model bias from BA predictions, improving robustness to unseen data. The corrected LBA per vertex and subject $LB{A^{\prime }}_{vs}$ is thus defined as:

$$LBA_{vs}^{\prime }=LBA_{vs}-\left(m_{\text{μ}}CA_{s}+b_{\text{μ}}\right)$$

In our analysis, we first bias corrected the ADNI CN cohort, giving us $m_{\text{μ}}^{CN}$ and $b_{\text{μ}}^{CN}$. We then bias corrected the ADNI AD cohort using these same coefficients $m_{\text{μ}}^{CN}$ and $b_{\text{μ}}^{CN}$ to avoid unintentionally removing disease-specific bias. This allowed us to compare across cohorts with respect to the same estimate and sample [54] of model bias. The difference in corrected LBAGs for ADs and CNs (AD - CN) therefore represents a vertex-wise difference map of disease-specific aging. We used this same procedure when comparing across sexes, where bias correction was performed for both cohorts using $m_{\text{μ}}^{CN}$ and $b_{\text{μ}}^{CN}$. Model losses were calculated *before* bias correction.

### Statistical significance testing.

After bias-correcting LBA estimates at each cortical vertex, we assessed statistical significance using region-averaged values per subject. Specifically, vertex-wise LBAs were averaged within each cortical region to yield regional LBAs for each subject. We then performed independent two-tailed t-tests per region to assess whether these regional LBAs deviated significantly from each subject’s CA, which allowed us to identify regions with evidence of advanced or delayed aging. When comparing across cohorts (e.g., controls vs. ADs), we conducted independent two-tailed t-tests to assess whether regional LBAs differed significantly between groups. All resulting p-values were corrected for multiple comparisons using the Benjamini-Hochberg procedure. Regions with adjusted p values ≥ 0.05 were deemed insignificant and masked during visualization (i.e., displayed in gray color).

### Sex comparisons.

To assess the model’s robustness across sexes, we compared LBAG differences between males and females within the CN cohort. To account for differences in CA distributions, which could be significant, we implemented a CA binning procedure. For each overlapping year of CA, one male and one female were randomly selected, and their LBAGs were recorded. This process was repeated 500 times to estimate the distribution of LBAGs across sexes (bootstrapping), enabling both global and local comparisons.

### Regressing cognitive scores.

To test whether higher BAGs were associated with poorer cognitive performance, we regressed BAGs against cognitive scores for each cohort. Trail Making Test B (TMT-B) scores equal to 300 were excluded to remove ceiling effects. The specific cognitive test measures analyzed were chosen from those available in ADNI, guided by prior BA work [19]. All scores were standardized and transformed so that higher values consistently indicated worse performance, making regression coefficients directly comparable across tests. We performed separate univariate linear regressions, with BAG as the dependent variable and the normalized cognitive score as the predictor of interest, while controlling for CA, sex, and years (y) of education. All p-values were corrected for multiple comparisons using the Benjamini–Hochberg procedure within each cohort.

## Results

### Model Performance.

The model was first evaluated using cross-validation (*N* = 13,146), yielding an average MAE of 7.56 y across all folds (range: 7.38 y – 7.72 y). We then retrained the model on the complete training set (*N* = 14,250) and generated predictions for the ADNI dataset for both the CN (MAE = 7.33 y) and AD (MAE = 8.15 y) cohorts.

### Comparing BAGs across cohorts.

**Figure 2.**
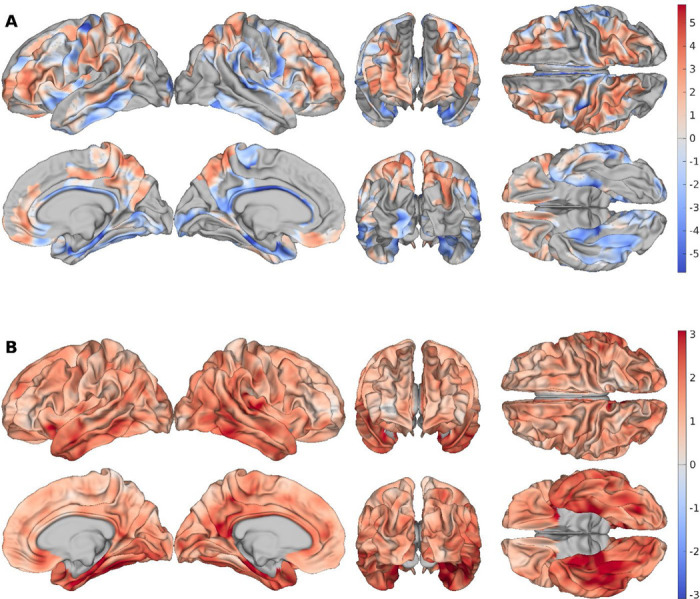
Bias-corrected LBAGs for ADNI CNs and ADNI ADs – ADNI CNs (A) Bias-corrected LBAGs for CN subjects. (B) Vertex-wise differences between AD and CN LBAGs (AD minus CN).

We next examined how BAGs were distributed across the cortical surface for both cohorts. After bias correction, GBAGs exhibited a mean of 0.00 y for the CNs, while ADs exhibited an average GBAG of 1.49 y (Figure S1). For LBAGs, in CNs, the largest effect sizes were observed in the prefrontal and parietal associations cortices (Figure 2A). When CNs were compared to ADs (AD–CN), the largest region-averaged group difference was observed in the parahippocampal gyrus (2.72 y), while the smallest was found in the lateral orbital sulcus (0.71 y). We also observed a cluster of similar LBAG differences (2.21 y to 2.34 y) occurring across several temporal regions, including the inferior temporal gyrus (2.21 y), temporal pole (2.28 y), planum polare (2.34 y), fusiform gyrus (2.30 y), and lateral occipito-temporal sulcus (2.27 y) (Figure 2B). ADNI AD LBAGs are shown in Figure S2. All regional LBAGs are available in **Tables S1–3** for the CNs, ADs, and AD-CNs respectively.

### Feature contributions.

**Figure 3.**
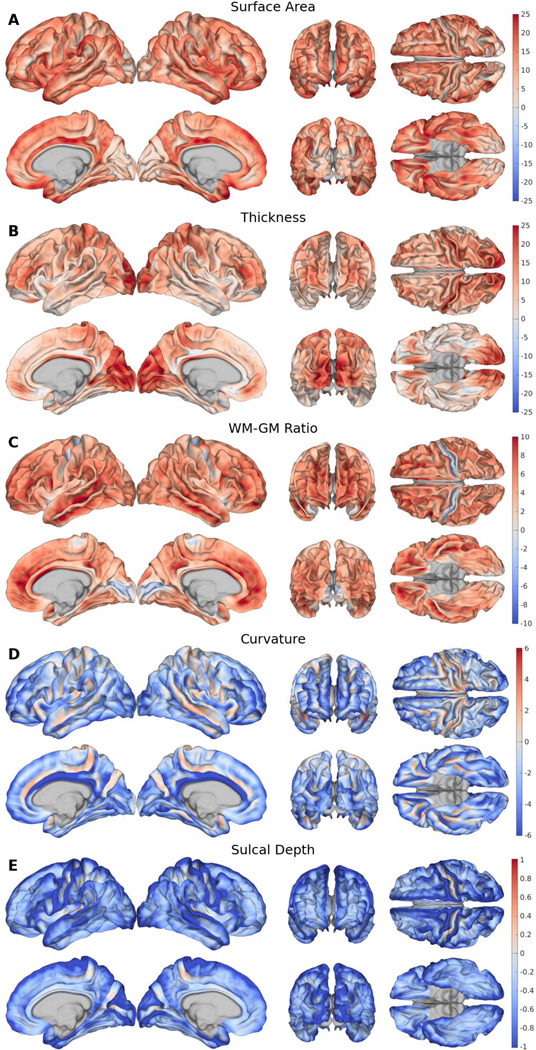
IGs for ADNI CN subjects. Group-level saliency maps showing the contribution of each cortical feature to model predictions: (A) SA, (B) CT, (C) GWR, (D) curvature, (E) sulcal depth. Saliency units retain relative significance across features.

Our saliency analysis using IGs revealed the local importance of each cortical feature in CNs (Figure 3). SA exhibited large saliency values across many cortical regions, most prominently on the crowns of gyri and throughout highly folded regions such as the inferior frontal gyrus, while the occipital lobe maintained weaker saliencies (Figure 3A). In contrast, CT displayed a highly localized effect, with strong saliencies clustered within the occipital lobe (Figure 3B). GWR exhibited its strongest effects, which were positive, in the frontal lobe and deep sulci, including the lateral, parieto-occipital, calcarine, and paracentral sulci (Figure 3C). Curvature presented a similar pattern of quantitative behavior, although shifted negatively, with positive saliencies localized to sulci, while gyri remained negative (Figure 3D). Sulcal depth exhibited weak, diffuse negative effects across the cortex, with only minor regional variation (Figure 3E). Among all features, CT and SA had the largest saliencies, while GWR, curvature, and sulcal depth exhibited progressively weaker saliencies. IGs were also calculated for ADs relative to CNs, but displayed little change (Figure S3). All regional LBAGs are available in **Tables S4–5** for the CNs and ADs respectively.

### BAGs predict cognitive scores.

**Figure 4.**
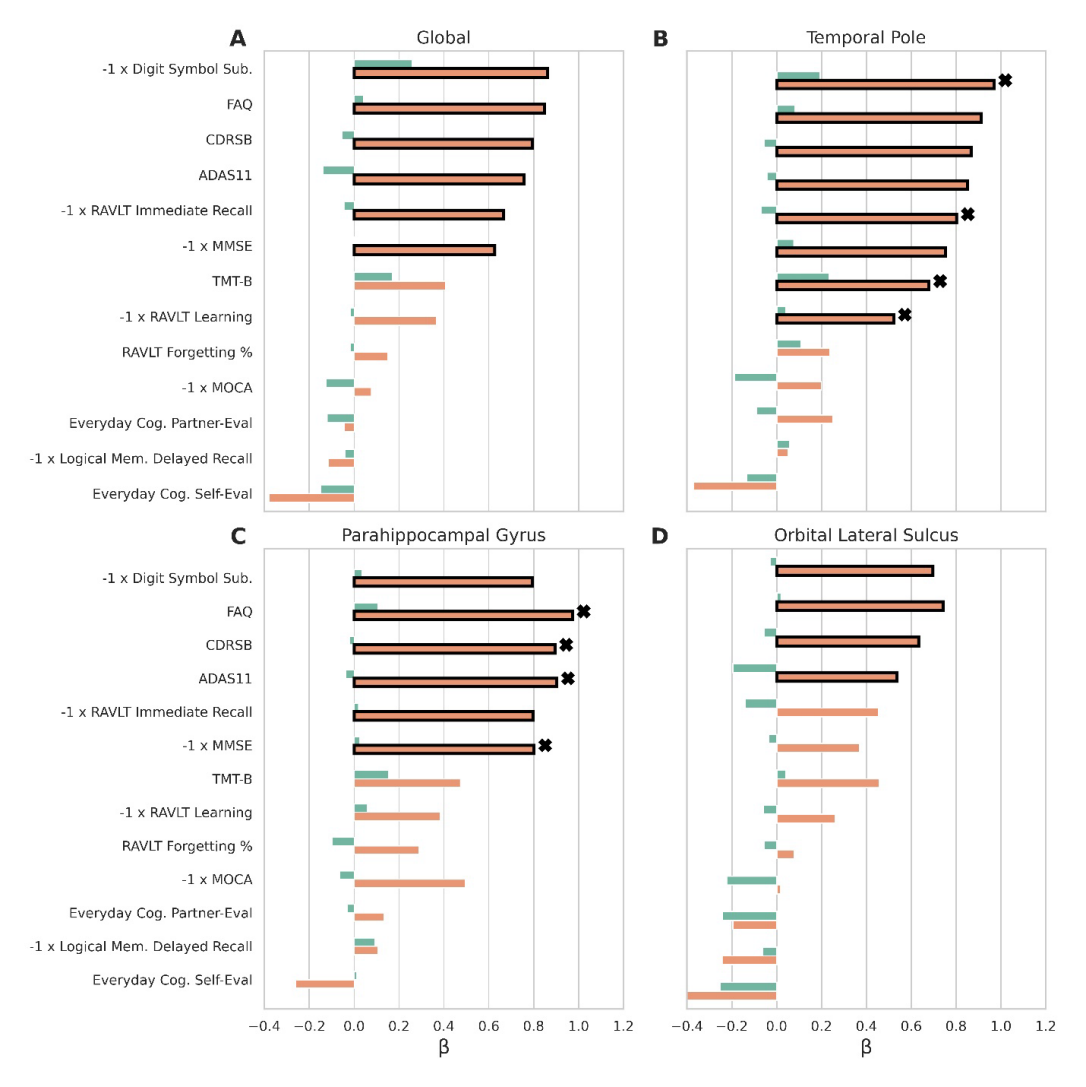
Regression results between BAG and cognitive scores. BAG was regressed against CA, cognitive score, sex, and years of education. Cognitive scores were z-score standardized and transformed (indicated by −1 x) such that a positive β coefficient indicates worsening performance with increasing BAGs for all tests. Bars show β coefficients for each test/cohort pair, using (A) GBAGs or regional LBAGs from the (B) temporal pole, (C) parahippocampal gyrus, and (D) orbital lateral sulcus. Green bars indicate regressions for the CN cohort, while orange bars indicate regressions for AD patients. Bars in bold indicate significant associations (adjusted p < 0.05). Among these, the largest significant coefficient for each test, across all regions, is additionally marked with an X.

Our regression analyses associating BAGs and cognitive scores revealed several structural-functional relationships. We performed regressions using BAGs averaged across the entire cortex (GBAGs), the temporal pole, the parahippocampal gyrus, and the orbital lateral sulcus. GBAGs were chosen as a proxy for global methods. The temporal pole was chosen due to its relevance in many high-level cognitive processes and neurodegenerative disorders**—**especially in a structural context [55–58]. The parahippocampal gyrus was selected because it exhibited the largest LBAG differences. The orbital lateral sulcus was chosen because it had the smallest LBAG differences. Across all regressions, no significant associations were observed between BAGs and cognitive scores for the CN cohort (Figure 4). In contrast, several cognitive tests were associated with both GBAGs and regional LBAGs for the AD cohort, surviving Benjamini-Hochberg correction. For GBAGs, these included Digit Symbol Substitution (*p* = 0.003), Functional Activities Questionnaire (FAQ; *p* < 1.0*x* × 10^−5^), Clinical Dementia Rating Sum of Boxes (CDRSB; *p* < 1.0 × 10^−4^), Alzheimer’s Disease Assessment Scale-11 (ADAS11; *p* < 1.0 × 10^−4^), Rey Auditory-Verbal Learning Test (RAVLT) immediate recall (*p* < 0.001), and Mini-Mental State Examination (MMSE; *p* < 0.001) scores (Figure 4A). Temporal pole LBAGs produced a similar pattern but with larger coefficients and additional associations with TMT-B (*p* < 0.05) and RAVLT learning (*p* < 0.05) scores (Figure 4B). The parahippocampal gyrus’ LBAGs had particularly strong associations with AD-relevant measures, such as FAQ (*p* < 1.0 × 10^−5^), CDRSB (*p* < 1.0 × 10^−5^), ADAS11 (*p* < 1.0 × 10^−5^), and MMSE (*p* < 1.0 × 10^−4^), with stronger associations than GBAGs for all tests except Digit Symbol Substitution (*p* = 0.01, Figure 4C). By contrast, the orbital lateral sulcus displayed weaker associations overall, and did not exhibit significant relationships with RAVLT immediate recall (*p* = 0.05) or MMSE (*p* = 0.09) scores (Figure 4D). Regression statistics are available in **Table S6**.

## Discussion

### Technical novelty.

Our LBA model addresses the limitations of GBA by modeling local aging patterns directly on the cortical surface. This enables explicit cross-regional comparisons, supporting downstream analysis of local aging patterns. Existing LBA methods leverage CNNs applied to volumetric data, where local neighborhoods are defined by sliding cubic kernels through the brain [26, 27]. Although effective for natural volumes, this Euclidean treatment disregards the folded topology of the cortical surface, risking geometric distortion that makes distant regions appear proximal [31–34]. We circumvent this problem by modeling the cortical surface in its native non-Euclidean topology, preserving vertex-level correspondence between input and output surfaces. This procedure addresses a key limitation of many learning approaches commonly applied in neuroimaging, which rely on regular grids or fixed volumetric neighborhoods and can distort local relationships when applied to data with irregular geometry and nonuniform anatomical connectivity. These design choices enable the detection of subtle, localized patterns that are often attenuated or lost in patch-based or heavily averaged analyses.

### Model performance and comparison to previous work.

Unlike CNN-based models, which rely on full-brain MRI intensities, our GNN focuses on intuitively meaningful morphometric features such as CT. In doing so, we constrain the amount of information to which our model is exposed, in exchange for heightened interpretability, greater spatial robustness, and reduced computational load. Even with this constraint, our model outperforms the original LBA model in terms of independent test-set MAE [26], though it slightly (< 0.7 y) underperforms more recent variants [27]. Additionally, our model successfully identified nuanced patterns of cortical aging, building upon existing literature in describing both CNs and ADs, with respect to both structure and cognition.

### Interpretation of structural findings.

Aging in CN individuals exhibited the largest LBAGs throughout the prefrontal and parietal association cortices. This supports prior literature, with these regions consistently being noted as early targets of aging-related morphometric change [59–61]. In particular, these findings recapitulate the ‘last-in first-out’ (LIFO) hypothesis, which postulates that cortical regions which develop later are the first to undergo atrophy in advanced age [59, 62, 63]. This is especially poignant in the context of the cortical surface, as regions which undergo greater SA expansion and exhibit pronounced folding, such as the prefrontal and association cortices, tend to reach maturity late into post-natal development [60, 62, 64–66]. This makes the LIFO hypothesis particularly relevant to our results, as our model directly leverages the morphometric features that underpin these trajectories.

A different pattern of aging was observed when comparing ADs to CNs, with the temporal lobe exhibiting the largest differences in LBAGs across cohorts, consistent with previous findings [67–69]. The parahippocampal gyrus, in particular, exhibited the most pronounced differences, exceeding other regions by a considerable margin (0.4 y). This aligns with prior AD literature establishing this region as the earliest and most atrophied across the cortical surface [3, 67, 70–74]. The parahippocampal gyrus shares strong functional connectivity with the hippocampus [75, 76], another area strongly implicated in AD [3, 67, 69], and supports critical cognitive processes including episodic memory and visuospatial processing [77]. These functions are characteristically impaired in AD [67, 78]. We concurrently observed a cluster of similar (2.21 y to 2.34 y) LBAG differences in other temporal regions, including the inferior temporal gyrus, temporal pole, planum polare, fusiform gyrus, and lateral occipito-temporal sulcus. These regions have all exhibited powerful associations with AD: the inferior temporal gyrus is known to undergo severe degeneration in AD [71, 79–81], and presents early atrophy that correlates with cognitive decline [81–83]. The temporal pole is among those regions earliest affected by AD [72, 84, 85], with cortical thinning in this region correlating with severity of tau pathology [56, 71, 86]. The planum polare displays significant and early gray matter atrophy as well as altered functional connectivity in AD [87–89]. Progressive atrophy of the fusiform gyrus is a reliable predictor of AD onset, with significant alterations observed across multiple domains throughout disease progression, including CT [90], volumetric changes [82, 91, 92], and epigenetic profiles [93]. Widening of the lateral occipito-temporal sulcus is characteristic of AD and is strongly discriminative between milder forms of CI and diagnosed AD, as well as between cognitive scores [9, 94, 95]. Taken together, these findings demonstrate that our model effectively captures AD-specific structural changes.

### Feature importance.

IGs revealed patterns of saliency indicating which features most strongly informed model predictions and where on the cortical surface these features were most influential. In CNs, SA and CT exhibited the largest net saliencies. SA, in particular, had large saliencies throughout the entire cortex, being most concentrated within the crowns of gyri and highly folded regions, and weakest in the occipital lobe. This lack of preference for the occipital lobe aligns with known aging patterns, where SA exhibits widespread changes particularly in the frontal, parietal, and temporal gyri [6, 60, 96]. Highly folded regions play a dominant role in multi-modal sensory networks as a function of their structure [97, 98], while simultaneously displaying elevated variability across genetically diverse populations [99–101]. Notably, regions with complex folding patterns tend to emerge later in development, once again concordant with the LIFO hypothesis [65, 66]. Similar trends are observed on gyral crowns, which exhibit substantial variability across populations that is driven by genetic factors [102–104], while being well-positioned as relay points for structural information [105, 106]. SA, as a whole, exhibits notable genetic covariance, revealing stronger associations with genetic profiles than other morphometric features [107, 108]. These findings support the potential of SA to enable the inference of genetic factors in a deep learning context. Because genetic factors, such as sex, contextualize how structural features change with age [101, 109, 110], SA’s heightened sensitivity to these factors [107, 108] predisposes it to capturing biologically meaningful variance. The same properties which make SA effective for supporting structural networks—its prominence in highly interconnected, information-dense regions—may likewise render it responsive to genetic influences that shape cortical reorganization. Thus, the saliency of SA likely reflects both its role as a conduit for genetic influences as well as its robustness in mediating large-scale structural networks across the cortex.

CT contrasted with SA, exhibiting saliencies that were largely constrained to the occipital lobe. The occipital lobe, though generally less vulnerable to aging effects than other lobes, demonstrates sharp reductions in CT in older adults, and at a far faster rate than in early- and middle- aged adults. This change in atrophy rate is exclusive to CT, with no corresponding acceleration observed in SA. Additionally, across all lobes of the brain, the occipital lobe exhibits the weakest correspondence between CT and SA [60, 111, 112], suggesting a decoupling of structural features which mirrors our saliency results.

GWR displayed positive saliencies in deep sulci and in the frontal lobe, with weaker effects observed elsewhere. This pattern aligns with known aging trajectories, where GWR exhibits greater decline in sulci than in gyri [10, 113, 114], particularly in the frontal lobe [10, 115, 116]. Notably, neither SA nor thickness express properties of WM, and it may be the case that this property makes GWR especially well-suited to modeling aging within the sulci, as sulcal morphology is intimately related to WM architecture [117, 118]. Prior research has suggested that differences in curvature lead to corresponding imbalances in WM and GM shrinkage, and vice-versa [12, 99, 119, 120], supporting a link between GWR and curvature that is substantiated by their overlapping saliencies within sulci. Sulcal depth exhibited the least informative saliencies, providing only weak, negative saliencies that were distributed relatively evenly throughout the cortex. This may be due to redundancy, where the information conveyed by sulcal depth is already being expressed by other features in the model. Cumulatively, these results provide insight into how distinct morphometric features capture complementary aspects of cortical aging.

### Relationship to cognition.

Our regression analyses confirmed that BAGs capture AD-specific cognitive impairment with high specificity. While GBAGs had significant associations with several cognitive scores known to decline with AD severity, LBAGs revealed additional associations that global measures failed to detect. Notably, the temporal pole exhibited significant associations with TMT-B and RAVLT learning scores that were not observed elsewhere. This aligns with prior work implicating the temporal pole in numerous high-level cognitive processes and neurodegenerative disorders [55–58]. Additionally, these cognitive associations supported our structural findings. The parahippocampal gyrus, which exhibited the largest differences in LBAGs across cohorts, correspondingly exhibited the strongest associations with cognitive measures most characteristic of AD progression (FAQ, CDRSB, ADAS11, MMSE) [121–124]. The temporal pole, which had significant but smaller β coefficients for these tests, likewise demonstrated notable AD-specific aging (i.e. increased LBAGs) that was attenuated relative to the parahippocampal gyrus. The orbital lateral sulcus, which had only weak cognitive associations, presented only weak increases in LBAGs. The subtlety of these findings demonstrates a concordance across structural and functional measures, with LBAGs grasping nuanced regional differences that were not expressed by GBAGs.

### Limitations of study design.

A key limitation of our study lies in the composition of the training and testing sets. The training set was skewed toward older adults and not fully age-matched, which may have reduced model generalizability. It was also sex-imbalanced (female-to-male ratio of 1.3:1) and, in the case of NACC, included multiple scans from the same participants to preserve the diversity of the training set (minimize the over-representation of UKBB). We tested the significance of potential sex-related biases explicitly by comparing the GBAGs (Figure S1) and LBAGs (**Table S7**) of males and females within the ADNI CN test set but found no significant differences. Another limitation pertains to the testing set, which only included older adults. This is appropriate for AD-focused analyses but prevents evaluation of model performance in younger populations. An additional limitation is that our feature analysis was conducted using IGs, which remain vulnerable to some of the drawbacks of saliency mentioned previously [23–25].

A more theoretical limitation of our approach stems from the geometric structure of the cortical surface, and how we leverage this to propagate information. From a graph-theoretic standpoint, the cortical mesh suffers from poor global connectivity, creating bottlenecks that impede the flow of information. This structural rigidity contributes to over-squashing, a well-documented phenomenon that hinders the model’s capacity to model long-range dependencies [125–127]. We observe this in our results: while the model was highly robust locally, it detected only modest GBAG differences between CN and AD cohorts (1.49 y). Various solutions to this problem have been proposed—most notably graph rewiring—but these come with significant trade-offs. Crucially, rewiring distorts the graph’s native topology, complicating anatomic interpretability. In addition, many of these techniques risk introducing over-smoothing, hindering local variability and potentially obscuring local brain-aging patterns [127–129]. In our own experiments, existing rewiring methods either failed to yield performance gains [130, 131], or were computationally infeasible due to the mesh’s scale [127, 132]. Future studies may benefit from the implementation of models that combine accurate local representations with more explicit multiscale organization. This could be achieved through hierarchical graph structures that facilitate information flow between fine- and coarse-scale descriptions, or by enriching graph representations to better capture long-range or cross-scale interactions beyond fixed local neighborhoods.

## Conclusions

We introduced a GNN for LBA estimation that leverages morphometric features to produce biologically grounded maps of cortical morphometric aging. The model identified the prefrontal and parietal association cortices as particularly vulnerable in normative aging, aligning with the LIFO hypothesis. IGs demonstrated that predictions were primarily driven by changes in SA (gyral crowns and highly folded regions) and CT (occipital lobes), with additional contributions from GWR (frontal lobes and sulcal troughs) and curvature (sulcal troughs), while sulcal depth was less useful. In patients with AD, we observed widespread patterns of accelerated aging, most prominently in the parahippocampal gyri and related temporal regions. Regressing LBAGs on cognitive scores recapitulated these findings, with core AD-related deficits being best captured by the parahippocampal gyrus. This analysis provided further insight into other regions as well, the temporal pole revealing subtle cognitive associations that global measures failed to detect, supporting the local sensitivity of our model. Cumulatively, our findings present and substantiate a framework for directly modelling cortical aging patterns as a function of morphology, supporting future application in both research and clinical contexts.

## Supplementary Material

Supplement 1

## Figures and Tables

**Table 1.** Dataset statistics. Number of scans $\left(N\right)$, descriptive statistics of chronological age (CA; minimum Min, maximum Max, mean μ, standard deviation σ), the male-to-female (M:F) ratio, and FreeSurfer version (FS version) used for MRI preprocessing.

			Chronological Age					
					
Repository	Set	N	Min	Max	μ	σ	M:F	FS version

UKBB	training	9,619	45.5	82.4	64.8	7.8	1:1.1	6.0.0
NACC	training	4,151	18.9	100.2	69.4	10.9	1:2.0	7.1.1
IXI	training	480	20.0	86.3	50.9	16.1	1:1.3	6.0.0

All	training	14,250	18.9	100.2	65.7	9.8	1:1.3	–

ADNI (CN)	testing	1,129	55.5	104.3	75.7	6.8	1:1.0	6.0.0
ADNI (AD)	testing	477	55.2	93.0	76.1	8.1	1:0.9	6.0.0

## References

[1] Wu B.S., , Cortical structure and the risk for Alzheimer’s disease: a bidirectional Mendelian randomization study. Transl Psychiatry, 2021. 11(1): p. 476. 34526483 10.1038/s41398-021-01599-x PMC8443658

[2] Bachmann T., , Longitudinal changes in surface based brain morphometry measures in amnestic mild cognitive impairment and Alzheimer’s Disease. Neuroimage Clin, 2023. 38: p. 103371. 10.1016/j.nicl.2023.103371 PMC10025277 36924681

[3] Chen L., , The impact of Alzheimer’s disease on cortical complexity and its underlying biological mechanisms. Brain Res Bull, 2025. 225: p. 111320. 10.1016/j.brainresbull.2025.111320 40189107

[4] Yuan P., Voelkle M.C., and Raz N., Fluid intelligence and gross structural properties of the cerebral cortex in middle-aged and older adults: A multi-occasion longitudinal study. Neuroimage, 2018. 172: p. 21–30. 29360573 10.1016/j.neuroimage.2018.01.032 PMC5910236

[5] Dai D., , Accurate prediction of AD patients using cortical thickness networks. Machine vision and applications, 2013. 24(7): p. 1445–1457.

[6] Nyberg L., Andersson M., and Lundquist A., Longitudinal change-change associations of cognition with cortical thickness and surface area. Aging Brain, 2023. 3: p. 100070. 10.1016/j.nbas.2023.100070 PMC10318300 37408792

[7] Bauer C.M., Cabral H.J., and Killiany R.J., Multimodal discrimination between normal aging, mild cognitive impairment and Alzheimer’s disease and prediction of cognitive decline. Diagnostics, 2018. 8(1): p. 14. 29415470 10.3390/diagnostics8010014 PMC5871997

[8] Shen X., , Variation in longitudinal trajectories of cortical sulci in normal elderly. Neuroimage, 2018. 166: p. 1–9. 29080713 10.1016/j.neuroimage.2017.10.010

[9] Sighinolfi G., , Sulcal Morphometry Predicts Mild Cognitive Impairment Conversion to Alzheimer’s Disease. J Alzheimers Dis, 2024. 99(1): p. 177–190. 38640154 10.3233/JAD-231192 PMC11191431

[10] Uribe C., , Gray/White Matter Contrast in Parkinson’s Disease. Front Aging Neurosci, 2018. 10: p. 89. 29636679 10.3389/fnagi.2018.00089 PMC5881246

[11] Jefferson A.L., , Gray & white matter tissue contrast differentiates Mild Cognitive Impairment converters from non-converters. Brain Imaging Behav, 2015. 9(2): p. 141–8. 24493370 10.1007/s11682-014-9291-2 PMC4146750

[12] Lin H.Y., , Differential Patterns of Gyral and Sulcal Morphological Changes During Normal Aging Process. Front Aging Neurosci, 2021. 13: p. 625931. 10.3389/fnagi.2021.625931 PMC7886979 33613271

[13] Apse R.R., , Morphometric Measurement of Mean Cortical Curvature: Analysis of Alterations in Cognitive Impairment. Medicina (Kaunas), 2025. 61(3). 10.3390/medicina61030531 PMC11944146 40142342

[14] Braak H., , Stages of the pathologic process in Alzheimer disease: age categories from 1 to 100 years. J Neuropathol Exp Neurol, 2011. 70(11): p. 960–9. 22002422 10.1097/NEN.0b013e318232a379

[15] Irimia A., , Statistical estimation of physiological brain age as a descriptor of senescence rate during adulthood. Brain Imaging Behav, 2015. 9(4): p. 678–89. 25376330 10.1007/s11682-014-9321-0 PMC4424195

[16] Amgalan A., , Brain age estimation reveals older adults’ accelerated senescence after traumatic brain injury. Geroscience, 2022. 44(5): p. 2509–2525. 35792961 10.1007/s11357-022-00597-1 PMC9768106

[17] Franke K., , Estimating the age of healthy subjects from T1-weighted MRI scans using kernel methods: exploring the influence of various parameters. Neuroimage, 2010. 50(3): p. 883–92. 20070949 10.1016/j.neuroimage.2010.01.005

[18] Elliott M.L., , Brain-age in midlife is associated with accelerated biological aging and cognitive decline in a longitudinal birth cohort. Molecular psychiatry, 2021. 26(8): p. 3829–3838. 31822815 10.1038/s41380-019-0626-7 PMC7282987

[19] Yin C., , Anatomically interpretable deep learning of brain age captures domain-specific cognitive impairment. Proc Natl Acad Sci U S A, 2023. 120(2): p. e2214634120. 10.1073/pnas.2214634120 PMC9926270 36595679

[20] Kalc P., , BrainAGE: Revisited and reframed machine learning workflow. Hum Brain Mapp, 2024. 45(3): p. e26632. 10.1002/hbm.26632 PMC10879910 38379519

[21] Lim H., , Brain Age Prediction Using Multi-Hop Graph Attention Combined with Convolutional Neural Network. Bioengineering (Basel), 2024. 11(3). 10.3390/bioengineering11030265 PMC10968402 38534539

[22] Sayres R., , Using a deep learning algorithm and integrated gradients explanation to assist grading for diabetic retinopathy. Ophthalmology, 2019. 126(4): p. 552–564. 30553900 10.1016/j.ophtha.2018.11.016

[23] Arun N., , Assessing the trustworthiness of saliency maps for localizing abnormalities in medical imaging. Radiology: Artificial Intelligence, 2021. 3(6): p. e200267. 10.1148/ryai.2021200267 PMC8637231 34870212

[24] Zhang J., , Revisiting the trustworthiness of saliency methods in radiology AI. Radiology: Artificial Intelligence, 2023. 6(1): p. e220221. 10.1148/ryai.220221 PMC10831523 38166328

[25] Venkatesh K., , Gradient-Based Saliency Maps Are Not Trustworthy Visual Explanations of Automated AI Musculoskeletal Diagnoses. Journal of Imaging Informatics in Medicine, 2024. 37(5): p. 2490–2499. 38710971 10.1007/s10278-024-01136-4 PMC11522229

[26] Popescu S.G., , Local Brain-Age: A U-Net Model. Front Aging Neurosci, 2021. 13: p. 761954. 10.3389/fnagi.2021.761954 PMC8710767 34966266

[27] Gianchandani N., A multitask deep learning model for voxel-level brain age estimation. in International Workshop on Machine Learning in Medical Imaging. 2023. Springer.

[28] Gaeta M., , T1 relaxation: Chemo-physical fundamentals of magnetic resonance imaging and clinical applications. Insights Imaging, 2024. 15(1): p. 200. 39120775 10.1186/s13244-024-01744-2 PMC11315875

[29] Van Leemput K., , Automated model-based tissue classification of MR images of the brain. IEEE Trans Med Imaging, 1999. 18(10): p. 897–908. 10628949 10.1109/42.811270

[30] Rawal A., , Recent advances in trustworthy explainable artificial intelligence: Status, challenges, and perspectives. IEEE Transactions on Artificial Intelligence, 2021. 3(6): p. 852–866.

[31] Masci J., Geodesic convolutional neural networks on riemannian manifolds. in Proceedings of the IEEE international conference on computer vision workshops. 2015.

[32] Zhao F., Spherical U-Net on cortical surfaces: methods and applications. in Information Processing in Medical Imaging: 26th International Conference, IPMI 2019, Hong Kong, China, June 2–7, 2019, Proceedings 26. 2019. Springer. 10.1007/978-3-030-20351-1_67 PMC7074928 32180666

[33] Bourlier A., Comparison between CNN and GNN pipelines for analysing the brain in development. in 20th International Conference on Computer Vision Theory and Applications. 2025. SCITEPRESS-Science and Technology Publications; SCITEPRESS.

[34] Tan J., , Application of improved graph convolutional network for cortical surface parcellation. Sci Rep, 2025. 15(1): p. 16409. 40355465 10.1038/s41598-025-00116-0 PMC12069630

[35] Li Z., , SurfGNN: A robust surface-based prediction model with interpretability for coactivation maps of spatial and cortical features. arXiv preprint arXiv:2411.05825, 2024. 10.1016/j.media.2025.103793 40934807

[36] Shin S.Y., , Deep vessel segmentation by learning graphical connectivity. Medical image analysis, 2019. 58: p. 101556. 31536906 10.1016/j.media.2019.101556

[37] Singh A., , Image Segmentation: Inducing graph-based learning. arXiv preprint arXiv:2501.03765, 2025.

[38] Xiao H., , GNNs surpass transformers in tumor medical image segmentation. Scientific Reports, 2025. 15(1): p. 19842. 40473649 10.1038/s41598-025-00002-9 PMC12141428

[39] Ronneberger O., Fischer P., and Brox T. U-net: Convolutional networks for biomedical image segmentation. in International Conference on Medical image computing and computer-assisted intervention. 2015. Springer.

[40] Beekly D.L., , The National Alzheimer’s Coordinating Center (NACC) database: the Uniform Data Set. Alzheimer Dis Assoc Disord, 2007. 21(3): p. 249–58. 17804958 10.1097/WAD.0b013e318142774e

[41] Kennedy D.N., , The NITRC image repository. Neuroimage, 2016. 124(Pt B): p. 1069–1073. 26044860 10.1016/j.neuroimage.2015.05.074 PMC4651733

[42] Littlejohns T.J., , The UK Biobank imaging enhancement of 100,000 participants: rationale, data collection, management and future directions. Nat Commun, 2020. 11(1): p. 2624. 32457287 10.1038/s41467-020-15948-9 PMC7250878

[43] Weiner M.W., , The Alzheimer’s Disease Neuroimaging Initiative: a review of papers published since its inception. Alzheimers Dement, 2013. 9(5): p. e111–94. 23932184 10.1016/j.jalz.2013.05.1769 PMC4108198

[44] Dale A.M., Fischl B., and Sereno M.I., Cortical surface-based analysis. I. Segmentation and surface reconstruction. Neuroimage, 1999. 9(2): p. 179–94. 9931268 10.1006/nimg.1998.0395

[45] Fischl B., , High-resolution intersubject averaging and a coordinate system for the cortical surface. Hum Brain Mapp, 1999. 8(4): p. 272–84. 10619420 10.1002/(SICI)1097-0193(1999)8:4<272::AID-HBM10>3.0.CO;2-4 PMC6873338

[46] Kipf T., Semi-supervised classification with graph convolutional networks. arXiv preprint arXiv:1609.02907, 2016.

[47] Ioffe S. and Szegedy C. Batch normalization: Accelerating deep network training by reducing internal covariate shift. in International conference on machine learning. 2015. pmlr.

[48] Kingma D.P., Adam: A method for stochastic optimization. arXiv preprint arXiv:1412.6980, 2014.

[49] Guo K.H., , Anatomic Interpretability in Neuroimage Deep Learning: Saliency Approaches for Typical Aging and Traumatic Brain Injury. Neuroinformatics, 2024. 22(4): p. 591–606. 39503843 10.1007/s12021-024-09694-2 PMC11579113

[50] Siegel N.T., , Explainable AI Methods for Neuroimaging: Systematic Failures of Common Tools, the Need for Domain-Specific Validation, and a Proposal for Safe Application. ArXiv, 2025.

[51] Sarasua I., Polsterl S., and Wachinger C., Hippocampal representations for deep learning on Alzheimer’s disease. Sci Rep, 2022. 12(1): p. 8619. 35597814 10.1038/s41598-022-12533-6 PMC9124220

[52] de Lange A.G. and Cole J.H., Commentary: Correction procedures in brain-age prediction. Neuroimage Clin, 2020. 26: p. 102229. 10.1016/j.nicl.2020.102229 PMC7049655 32120292

[53] Beheshti I., , Bias-adjustment in neuroimaging-based brain age frameworks: A robust scheme. Neuroimage Clin, 2019. 24: p. 102063. 10.1016/j.nicl.2019.102063 PMC6861562 31795063

[54] Zhang B., , Age-level bias correction in brain age prediction. Neuroimage Clin, 2023. 37: p. 103319. 10.1016/j.nicl.2023.103319 PMC9860514 36634514

[55] Pehrs C., , The temporal pole top-down modulates the ventral visual stream during social cognition. Cerebral Cortex, 2017. 27(1): p. 777–792. 26604273 10.1093/cercor/bhv226

[56] Herlin B., Navarro V., and Dupont S., The temporal pole: From anatomy to function-A literature appraisal. J Chem Neuroanat, 2021. 113: p. 101925. 10.1016/j.jchemneu.2021.101925 33582250

[57] Guo P., , Associations of Neurocognition and Social Cognition With Brain Structure and Function in Early-Onset Schizophrenia. Front Psychiatry, 2022. 13: p. 798105. 10.3389/fpsyt.2022.798105 PMC8866448 35222115

[58] Rostowsky K.A., Irimia A., and Alzheimer’s Disease Neuroimaging I., Acute cognitive impairment after traumatic brain injury predicts the occurrence of brain atrophy patterns similar to those observed in Alzheimer’s disease. Geroscience, 2021. 43(4): p. 2015–2039. 33900530 10.1007/s11357-021-00355-9 PMC8492819

[59] McGinnis S.M., , Age-related changes in the thickness of cortical zones in humans. Brain Topogr, 2011. 24(3–4): p. 279–91. 21842406 10.1007/s10548-011-0198-6 PMC3600370

[60] Hogstrom L.J., , The structure of the cerebral cortex across adult life: age-related patterns of surface area, thickness, and gyrification. Cereb Cortex, 2013. 23(11): p. 2521–30. 22892423 10.1093/cercor/bhs231

[61] Madan C.R., Age-related decrements in cortical gyrification: Evidence from an accelerated longitudinal dataset. Eur J Neurosci, 2021. 53(5): p. 1661–1671. 33171528 10.1111/ejn.15039 PMC7979529

[62] Douaud G., , A common brain network links development, aging, and vulnerability to disease. Proc Natl Acad Sci U S A, 2014. 111(49): p. 17648–53. 25422429 10.1073/pnas.1410378111 PMC4267352

[63] Yeatman J.D., Wandell B.A., and Mezer A.A., Lifespan maturation and degeneration of human brain white matter. Nat Commun, 2014. 5: p. 4932. 25230200 10.1038/ncomms5932 PMC4238904

[64] Hill J., , Similar patterns of cortical expansion during human development and evolution. Proc Natl Acad Sci U S A, 2010. 107(29): p. 13135–40. 20624964 10.1073/pnas.1001229107 PMC2919958

[65] Dubois J., , The dynamics of cortical folding waves and prematurity-related deviations revealed by spatial and spectral analysis of gyrification. Neuroimage, 2019. 185: p. 934–946. 29522888 10.1016/j.neuroimage.2018.03.005

[66] Voorhies W.I., , Cognitive insights from tertiary sulci in prefrontal cortex. Nature Communications, 2021. 12(1): p. 5122. 10.1038/s41467-021-25162-w PMC8387420 34433806

[67] Pini L., , Brain atrophy in Alzheimer’s Disease and aging. Ageing Res Rev, 2016. 30: p. 25–48. 26827786 10.1016/j.arr.2016.01.002

[68] de Flores R., , Medial Temporal Lobe Networks in Alzheimer’s Disease: Structural and Molecular Vulnerabilities. J Neurosci, 2022. 42(10): p. 2131–2141. 35086906 10.1523/JNEUROSCI.0949-21.2021 PMC8916768

[69] Migliaccio R. and Cacciamani F., The temporal lobe in typical and atypical Alzheimer disease. Handb Clin Neurol, 2022. 187: p. 449–466. 35964987 10.1016/B978-0-12-823493-8.00004-3

[70] Bakkour A., Morris J.C., and Dickerson B.C., The cortical signature of prodromal AD: regional thinning predicts mild AD dementia. Neurology, 2009. 72(12): p. 1048–55. 19109536 10.1212/01.wnl.0000340981.97664.2f PMC2677470

[71] Dickerson B.C., , The cortical signature of Alzheimer’s disease: regionally specific cortical thinning relates to symptom severity in very mild to mild AD dementia and is detectable in asymptomatic amyloid-positive individuals. Cereb Cortex, 2009. 19(3): p. 497–510. 18632739 10.1093/cercor/bhn113 PMC2638813

[72] Verfaillie S.C., , Thinner temporal and parietal cortex is related to incident clinical progression to dementia in patients with subjective cognitive decline. Alzheimers Dement (Amst), 2016. 5: p. 43–52. 28054027 10.1016/j.dadm.2016.10.007 PMC5198882

[73] Krajcovicova L., Klobusiakova P., and Rektorova I., Gray Matter Changes in Parkinson’s and Alzheimer’s Disease and Relation to Cognition. Curr Neurol Neurosci Rep, 2019. 19(11): p. 85. 31720859 10.1007/s11910-019-1006-z PMC6854046

[74] Dzianok P., , Cortical thinning in temporal pole, a core region in Alzheimer’s disease, in non-demented, middle-aged APOE-ε4 and PICALM-AA/AG carriers. bioRxiv, 2025: p. 2025.02. 21.639542.

[75] Libby L.A., , Differential connectivity of perirhinal and parahippocampal cortices within human hippocampal subregions revealed by high-resolution functional imaging. Journal of Neuroscience, 2012. 32(19): p. 6550–6560. 22573677 10.1523/JNEUROSCI.3711-11.2012 PMC3374643

[76] Maller J.J., , Revealing the Hippocampal Connectome through Super-Resolution 1150-Direction Diffusion MRI. Sci Rep, 2019. 9(1): p. 2418. 30787303 10.1038/s41598-018-37905-9 PMC6382767

[77] Aminoff E.M., Kveraga K., and Bar M., The role of the parahippocampal cortex in cognition. Trends Cogn Sci, 2013. 17(8): p. 379–90. 23850264 10.1016/j.tics.2013.06.009 PMC3786097

[78] Association., A.P., Diagnostic and statistical manual of mental disorders (5th ed.). 5 ed. 2013: American Psychiatric Association Publishing.

[79] Scheff S.W., , Synaptic loss in the inferior temporal gyrus in mild cognitive impairment and Alzheimer’s disease. J Alzheimers Dis, 2011. 24(3): p. 547–57. 21297265 10.3233/JAD-2011-101782 PMC3098316

[80] Sattari N., , Assessing the Changes of Cortical Thickness in Alzheimer Disease With MRI Using Freesurfer Software. Basic Clin Neurosci, 2022. 13(2): p. 185–192. 36425945 10.32598/bcn.2021.1779.1 PMC9682320

[81] Wuestefeld A., , Tau, atrophy, and domain-specific cognitive impairment in typical Alzheimer’s disease. Alzheimers Dement, 2025. 21(7): p. e70511. 10.1002/alz.70511 PMC12290488 40709494

[82] Convit A., , Atrophy of the medial occipitotemporal, inferior, and middle temporal gyri in non-demented elderly predict decline to Alzheimer’s disease☆. Neurobiology of aging, 2000. 21(1): p. 19–26. 10794844 10.1016/s0197-4580(99)00107-4

[83] Lu F., , The Correlations between Volume Loss of Temporal and Subcortical Functional Subregions and Cognitive Impairment at Various Stages of Cognitive Decline. J Integr Neurosci, 2024. 23(12): p. 220. 39735962 10.31083/j.jin2312220

[84] Arnold S.E., , The topographical and neuroanatomical distribution of neurofibrillary tangles and neuritic plaques in the cerebral cortex of patients with Alzheimer’s disease. Cereb Cortex, 1991. 1(1): p. 103–16. 1822725 10.1093/cercor/1.1.103

[85] Arnold S.E., Hyman B.T., and Van Hoesen G.W., Neuropathologic changes of the temporal pole in Alzheimer’s disease and Pick’s disease. Arch Neurol, 1994. 51(2): p. 145–50. 8304839 10.1001/archneur.1994.00540140051014

[86] LaPoint M.R., , The association between tau PET and retrospective cortical thinning in clinically normal elderly. Neuroimage, 2017. 157: p. 612–622. 28545932 10.1016/j.neuroimage.2017.05.049 PMC5772972

[87] Rasero J., , Multivariate regression analysis of structural MRI connectivity matrices in Alzheimer’s disease. PLoS One, 2017. 12(11): p. e0187281. 10.1371/journal.pone.0187281 PMC5685585 29135998

[88] Lenhart L., , Anatomically Standardized Detection of MRI Atrophy Patterns in Early-Stage Alzheimer’s Disease. Brain Sciences, 2021. 11(11): p. 1491. 34827490 10.3390/brainsci11111491 PMC8615991

[89] Stocks J., , Spatial and Temporal Progression of Neurodegeneration in Confirmed and Suspected TDP-43 Type C Pathology. Imaging Neuroscience, 2025. 10.1162/IMAG.a.83 PMC12320738 40761602

[90] Yang H., , Study of brain morphology change in Alzheimer’s disease and amnestic mild cognitive impairment compared with normal controls. General psychiatry, 2019. 32(2): p. e100005. 10.1136/gpsych-2018-100005 PMC6551438 31179429

[91] Halliday G., , Identifying severely atrophic cortical subregions in Alzheimer’s disease. Neurobiology of aging, 2003. 24(6): p. 797–806. 12927762 10.1016/s0197-4580(02)00227-0

[92] Convit A., , Specific hippocampal volume reductions in individuals at risk for Alzheimer’s disease. Neurobiology of aging, 1997. 18(2): p. 131–138. 9258889 10.1016/s0197-4580(97)00001-8

[93] Ma D., , The fusiform gyrus exhibits an epigenetic signature for Alzheimer’s disease. Clinical epigenetics, 2020. 12(1): p. 129. 32854783 10.1186/s13148-020-00916-3 PMC7457273

[94] Ficiarà E., Predicting progression from mild cognitive impairment to Alzheimer’s disease using MRI-based cortical features and a two-state Markov Model. in 2021 IEEE 18th International Symposium on Biomedical Imaging (ISBI). 2021. IEEE. 10.1109/isbi48211.2021.9434143 PMC8935949 35321154

[95] Mortamais M., , Sulcal morphology as cognitive decline predictor in older adults with memory complaints. Neurobiology of Aging, 2022. 113: p. 84–94. 35325814 10.1016/j.neurobiolaging.2022.02.003

[96] Yu J., Age-related decline in thickness and surface area in the cortical surface and hippocampus: lifespan trajectories and decade-by-decade analyses. Geroscience, 2024. 46(6): p. 6213–6227. 38831181 10.1007/s11357-024-01220-1 PMC11494012

[97] Bassett D.S., , Hierarchical organization of human cortical networks in health and schizophrenia. J Neurosci, 2008. 28(37): p. 9239–48. 18784304 10.1523/JNEUROSCI.1929-08.2008 PMC2878961

[98] He Y. and Evans A., Graph theoretical modeling of brain connectivity. Curr Opin Neurol, 2010. 23(4): p. 341–50. 20581686 10.1097/WCO.0b013e32833aa567

[99] Liu T., , The effects of age and sex on cortical sulci in the elderly. Neuroimage, 2010. 51(1): p. 19–27. 20156569 10.1016/j.neuroimage.2010.02.016

[100] Ronan L. and Fletcher P.C., From genes to folds: a review of cortical gyrification theory. Brain Struct Funct, 2015. 220(5): p. 2475–83. 25511709 10.1007/s00429-014-0961-z PMC4549381

[101] Alexander-Bloch A.F., , Imaging local genetic influences on cortical folding. Proc Natl Acad Sci U S A, 2020. 117(13): p. 7430–7436. 32170019 10.1073/pnas.1912064117 PMC7132284

[102] Akula S.K., Exposito-Alonso D., and Walsh C.A., Shaping the brain: The emergence of cortical structure and folding. Dev Cell, 2023. 58(24): p. 2836–2849. 38113850 10.1016/j.devcel.2023.11.004 PMC10793202

[103] Huang Y., , Genetic Influence on Gyral Peaks. Neuroimage, 2023. 280: p. 120344. 10.1016/j.neuroimage.2023.120344 37619794

[104] Cao G., , Gyral peak variations between HCP and CHCP: functional and structural implications. Brain Struct Funct, 2025. 230(2): p. 37. 39903275 10.1007/s00429-025-02894-9

[105] Zhang S., , Gyral peaks: Novel gyral landmarks in developing macaque brains. Hum Brain Mapp, 2022. 43(15): p. 4540–4555. 35713202 10.1002/hbm.25971 PMC9491295

[106] Zhang S., , Gyral peaks and patterns in human brains. Cereb Cortex, 2023. 33(11): p. 6708–6722. 36646465 10.1093/cercor/bhac537 PMC10422926

[107] Panizzon M.S., , Distinct genetic influences on cortical surface area and cortical thickness. Cereb Cortex, 2009. 19(11): p. 2728–35. 19299253 10.1093/cercor/bhp026 PMC2758684

[108] Grasby K.L., , The genetic architecture of the human cerebral cortex. Science, 2020. 367(6484). 10.1126/science.aay6690 PMC7295264 32193296

[109] Cui D., , Age-and sex-related differences in cortical morphology and their relationships with cognitive performance in healthy middle-aged and older adults. Quantitative Imaging in Medicine and Surgery, 2022. 13(2): p. 1083. 36819243 10.21037/qims-22-583 PMC9929420

[110] Irimia A., Cross-Sectional Volumes and Trajectories of the Human Brain, Gray Matter, White Matter and Cerebrospinal Fluid in 9473 Typically Aging Adults. Neuroinformatics, 2021. 19(2): p. 347–366. 32856237 10.1007/s12021-020-09480-w PMC7910325

[111] van Velsen E.F., , Brain cortical thickness in the general elderly population: the Rotterdam Scan Study. Neurosci Lett, 2013. 550: p. 189–94. 23831346 10.1016/j.neulet.2013.06.063

[112] Storsve A.B., , Differential longitudinal changes in cortical thickness, surface area and volume across the adult life span: regions of accelerating and decelerating change. J Neurosci, 2014. 34(25): p. 8488–98. 24948804 10.1523/JNEUROSCI.0391-14.2014 PMC6608217

[113] Salat D.H., , Hippocampal degeneration is associated with temporal and limbic gray matter/white matter tissue contrast in Alzheimer’s disease. Neuroimage, 2011. 54(3): p. 1795–1802. 20965261 10.1016/j.neuroimage.2010.10.034 PMC3021138

[114] Putcha D., , Gray to white matter signal ratio as a novel biomarker of neurodegeneration in Alzheimer’s disease. NeuroImage: Clinical, 2023. 37: p. 103303. 10.1016/j.nicl.2022.103303 PMC9830315 36586361

[115] Salat D.H., , Age-associated alterations in cortical gray and white matter signal intensity and gray to white matter contrast. Neuroimage, 2009. 48(1): p. 21–8. 19580876 10.1016/j.neuroimage.2009.06.074 PMC2750073

[116] Kong L., , Reduced gray to white matter tissue intensity contrast in schizophrenia. PLoS One, 2012. 7(5): p. e37016. 10.1371/journal.pone.0037016 PMC3352852 22615876

[117] Van Essen D.C., A 2020 view of tension-based cortical morphogenesis. Proceedings of the National Academy of Sciences, 2020. 117(52): p. 32868–32879. 10.1073/pnas.2016830117 PMC7780001 33323481

[118] Maboudian S.A., , Defining Overlooked Structures Reveals New Associations between the Cortex and Cognition in Aging and Alzheimer’s Disease. J Neurosci, 2024. 44(16). 10.1523/JNEUROSCI.1714-23.2024 PMC11026365 38383497

[119] Deppe M., , Increased cortical curvature reflects white matter atrophy in individual patients with early multiple sclerosis. Neuroimage Clin, 2014. 6: p. 475–87. 25610761 10.1016/j.nicl.2014.02.012 PMC4299934

[120] Demirci N. and Holland M.A., Cortical thickness systematically varies with curvature and depth in healthy human brains. Hum Brain Mapp, 2022. 43(6): p. 2064–2084. 35098606 10.1002/hbm.25776 PMC8933257

[121] Teng E., , Utility of the functional activities questionnaire for distinguishing mild cognitive impairment from very mild Alzheimer disease. Alzheimer Disease & Associated Disorders, 2010. 24(4): p. 348–353. 20592580 10.1097/WAD.0b013e3181e2fc84 PMC2997338

[122] Coley N., , Suitability of the Clinical Dementia Rating-Sum of Boxes as a single primary endpoint for Alzheimer’s disease trials. Alzheimer’s & Dementia, 2011. 7(6): p. 602–610. e2. 10.1016/j.jalz.2011.01.005 21745761

[123] Kueper J.K., Speechley M., and Montero-Odasso M., The Alzheimer’s disease assessment scale–cognitive subscale (ADAS-Cog): modifications and responsiveness in pre-dementia populations. a narrative review. Journal of Alzheimer’s Disease, 2018. 63(2): p. 423–444. 10.3233/JAD-170991 PMC5929311 29660938

[124] Benoit J.S., , Longitudinal sensitivity of Alzheimer’s disease severity staging. American Journal of Alzheimer’s Disease & Other Dementias^®^, 2020. 35: p. 1533317520918719. 10.1177/1533317520918719 PMC10624049 32573256

[125] Alon U. and Yahav E., On the bottleneck of graph neural networks and its practical implications. arXiv preprint arXiv:2006.05205, 2020.

[126] Deac A., Lackenby M., and Veličković P. Expander graph propagation. in Learning on Graphs Conference. 2022. PMLR.

[127] Wilson J., Bechler-Speicher M., and Veličković P., Cayley graph propagation. arXiv preprint arXiv:2410.03424, 2024.

[128] Barbero F., , Locality-aware graph-rewiring in gnns. arXiv preprint arXiv:2310.01668, 2023.

[129] Attali H., Buscaldi D., and Pernelle N., Rewiring Techniques to Mitigate Oversquashing and Oversmoothing in GNNs: A Survey. arXiv preprint arXiv:2411.17429, 2024.

[130] Gilmer J., Neural message passing for quantum chemistry. in International conference on machine learning. 2017. PMLR.

[131] Qian C., , Probabilistic graph rewiring via virtual nodes. arXiv preprint arXiv:2405.17311, 2024.

[132] Ni C.-C., , Community detection on networks with Ricci flow. Scientific reports, 2019. 9(1): p. 9984. 31477786 10.1038/s41598-019-49491-5 PMC6718387

