# Supplementary material for: Graph Neural Network Reveals the Local Cortical Morphology of Brain Aging in Normal Cognition and Alzheimer’s Disease
Source: ArXiv. 2026 Jan 23:arXiv:2601.10912v4. Preprint. [Version 4] (PMC12869401)
Supplement: Supplement 1 [file NIHPP2601.10912v4-supplement-1.pdf]

Supplementary Figures

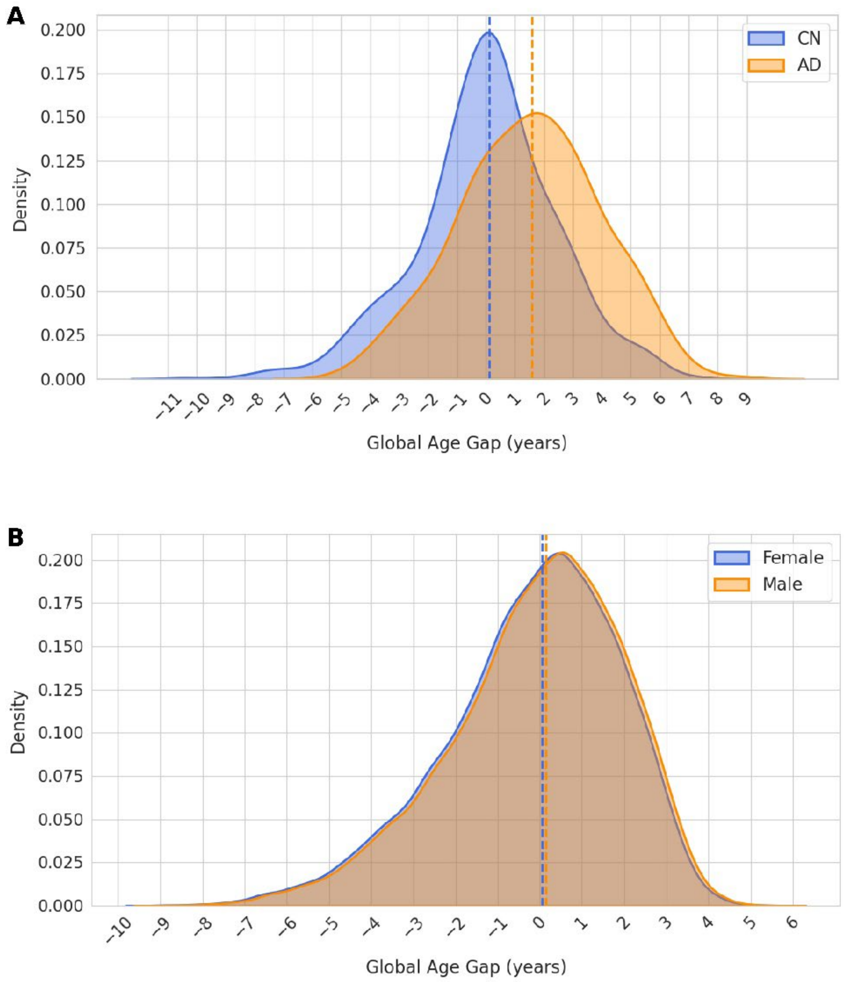

Supplementary Figure 1. Distribution of GBAGs for the ADNI test sets. (A) Distribution of GBAGs for the ADNI CN and ADNI AD test sets. Dashed lines represent the medians of each group. CN subjects: mean = 0.00 y, median = 0.11 y, std = 2.44 y, var = 5.93 y; AD subjects: mean = 1.49 y, median = 1.60 y, std = 2.46 y, var = 6.05 y. (B) Male and female samples were randomly selected using age-binned matching of corrected AGs, with this procedure repeated 500 times (bootstrapped) to generate group distributions. The resulting GBAG distributions for males and females are nearly identical. CN subjects: mean = 0.00 y, median = 0.11 y, std = 2.44 y, var = 5.93 y; AD subjects: mean = 1.49 y, median = 1.60 y, std = 2.46 y, var = 6.05 y.

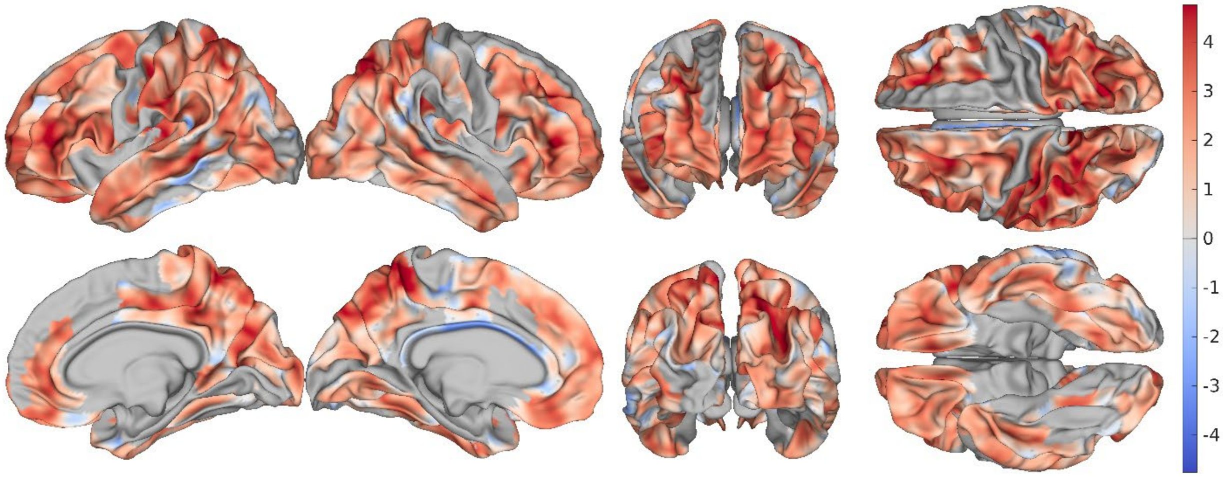

Supplementary Figure 2. Bias-corrected LBAGs for the ADNI AD test set. Gray regions indicate non-significant vertices.

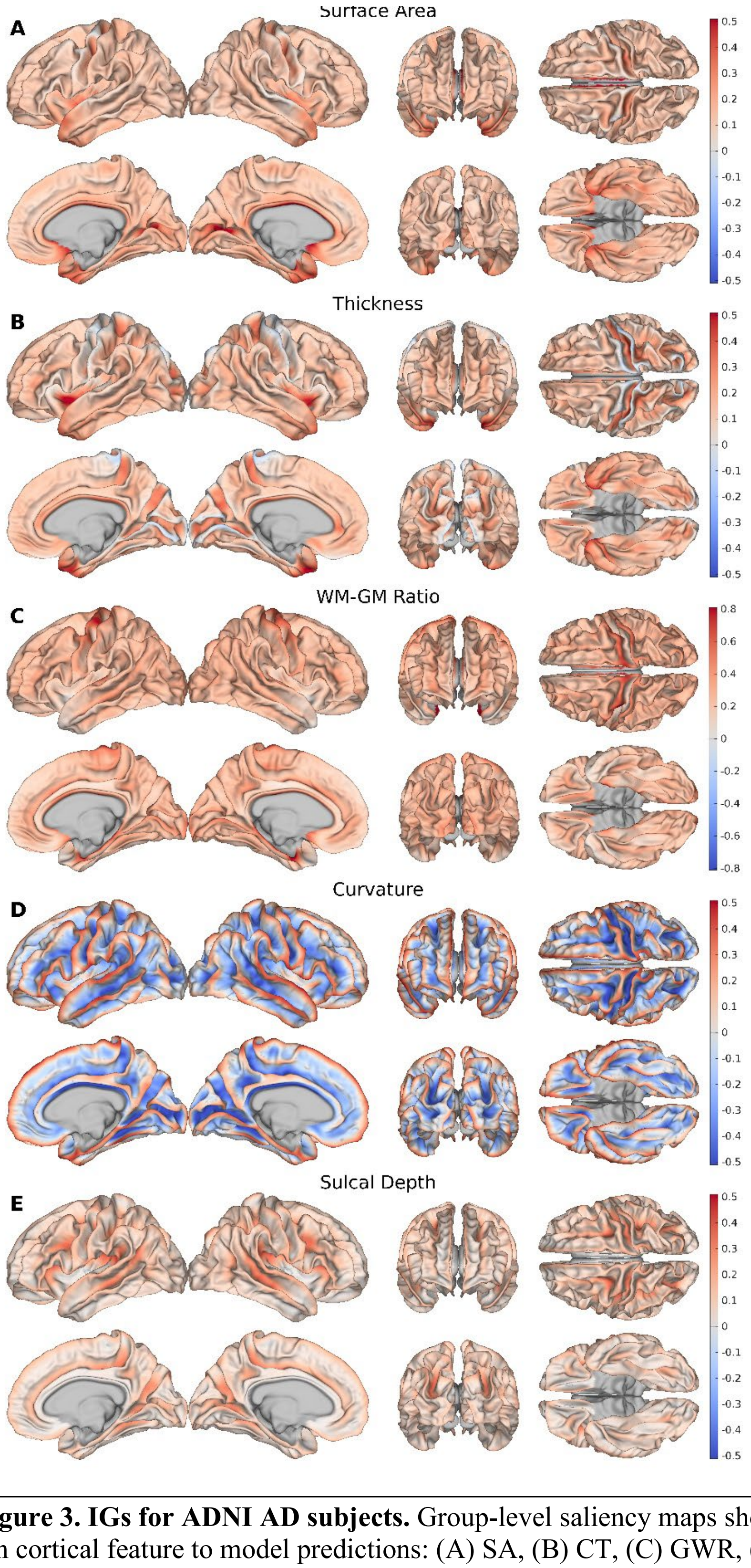

Supplementary Figure 3. IGs for ADNI AD subjects. Group-level saliency maps showing the contribution of each cortical feature to model predictions: (A) SA, (B) CT, (C) GWR, (D) curvature, (E) sulcal depth. Saliency units retain relative magnitude across features. Only small differences in saliency were observed.
